# Prognostic Role of Programmed Death Ligand-1 on Tumor-Infiltrating Immune Cells in “High-Risk” Patients Following Radical Cystectomy: A Retrospective Cohort Study

**DOI:** 10.3389/fonc.2021.706503

**Published:** 2021-08-20

**Authors:** Chung Un Lee, Dong Hyeon Lee, Wan Song

**Affiliations:** ^1^Department of Urology, Samsung Medical Center, Sungkyunkwan University School of Medicine, Seoul, South Korea; ^2^Department of Urology, Ewha Womans University Medical Center, Ewha Womans University School of Medicine, Seoul, South Korea

**Keywords:** high-risk, programmed death ligand-1, radical cystectomy, recurrence, tumor-infiltrating immune cell

## Abstract

**Purpose:**

The aim of this study is to investigate the prognostic role of programmed death ligand-1 (PD-L1) on tumor-infiltrating immune cells (TIICs) in patients after radical cystectomy (RC) for bladder cancer (BCa).

**Materials and Methods:**

We retrospectively reviewed 92 “high-risk” (≥pT3a and/or pN+) patients who underwent RC for BCa, without adjuvant chemotherapy (AC), between April 2014 and December 2019. PD-L1 on TIICs was measured only using the VENTANA (SP-142) immunohistochemistry assay. Patients were categorized into three groups based to the percentage of the tumor area covered by PD-L1 on TIICs: IC0 (<1%), IC1 (≥1% and <5%), and IC2/3 (≥5%). Positive PD-L1 was defined as IC2/3 (≥5%). Kaplan–Meier survival analysis was used to illustrate recurrence-free survival (RFS), and Cox proportional hazard models were used to identify predictive factors of tumor recurrence.

**Results:**

Within the cohort, the proportions of PD-L1 IC0, IC1, and IC2/3 were 21.7%, 23.9%, and 54.4%, respectively. At follow-up (mean 31.3 months), tumor recurrence was identified in 49 patients (53.3%). Using multivariable analysis, tumor stage (pT4; *P*=0.005), positive lymph nodes (*P*=0.021), and positive PD-L1 on TIICs (*P*=0.010) were independent predictors of tumor recurrence. The 2- and 3-year RFS rates were 67.7% and 64.2% in negative PD-L1 on TIICs, while 27.8% and 22.3% in positive PD-L1 on TIICs, respectively.

**Conclusions:**

Positive PD-L1 on TIICs was significantly associated with poorer RFS in “high-risk” patients after RC without AC. Our results support the use of adjuvant immunotherapy in “high-risk” patients with positive PD-L1 on TIICs after RC.

## Introduction

Currently, the guidelines of European Association of Urology (EAU) on muscle-invasive bladder cancer (MIBC) recommend cisplatin-based combination adjuvant chemotherapy (AC) after radical cystectomy (RC) in high-risk patients (≥pT3a and/or pN+) if they did not receive neoadjuvant chemotherapy (NAC) (1). However, approximately half of patients with advanced bladder cancer (BCa) are not cisplatin-eligible because of comorbidities such as creatinine clearance < 60 mL/min, Eastern Cooperative Oncology Group (ECOG) performance status ≥ 2, New York Heart Association (NYHA) class 3 heart failure, and grade ≥ 2 neuropathy (2–4). In addition, about a third of patients only experience adverse events related to AC without treatment benefits (5). For this reason, a novel strategy using immune checkpoint inhibitors is emerging as a promising therapeutic approach because of their relatively lower toxicities compared to chemotherapy (6, 7).

To date, because of its relatively high tumor mutational burden, BCa is regarded as an immunogenic tumor (8, 9). Blocking of programmed death-1 (PD-1)/programmed death ligand-1 (PD-L1) interaction has revealed positive results in BCa by restoring T cell-mediated immune responses (10, 11). As a result, three phase III trials are ongoing to identify the efficacy of atezolizumab (IMvigor010 or NCT0245033) (12), nivolumab (CheckMate274 or NCT02632409) (13) and pembrolizumab (AMBASSADOR or NCT03244384) (14) in the adjuvant setting following RC. However, there are few studies on predictive biomarkers that can be used to choose patients suitable for adjuvant immunotherapy.

Therefore, in this study, we examined PD-L1 on tumor-infiltrating immune cells (TIICs) in RC specimens to investigate the prognostic role of PD-L1 on TIICs as a predictive biomarker by analyzing the correlation with tumor recurrence in “high-risk” patients after RC.

## Materials and Methods

### Patients’ Selection

This study was approved by the Institutional Review Board of Ewha Womans University Mokdong Hospital (IRB No. 2019-02-004) and performed in accordance with the Declaration of Helsinki. Informed consent of patients was waived owing to the study design, but all patient data complied with relevant privacy regulations and data protection

**Figure 1:**
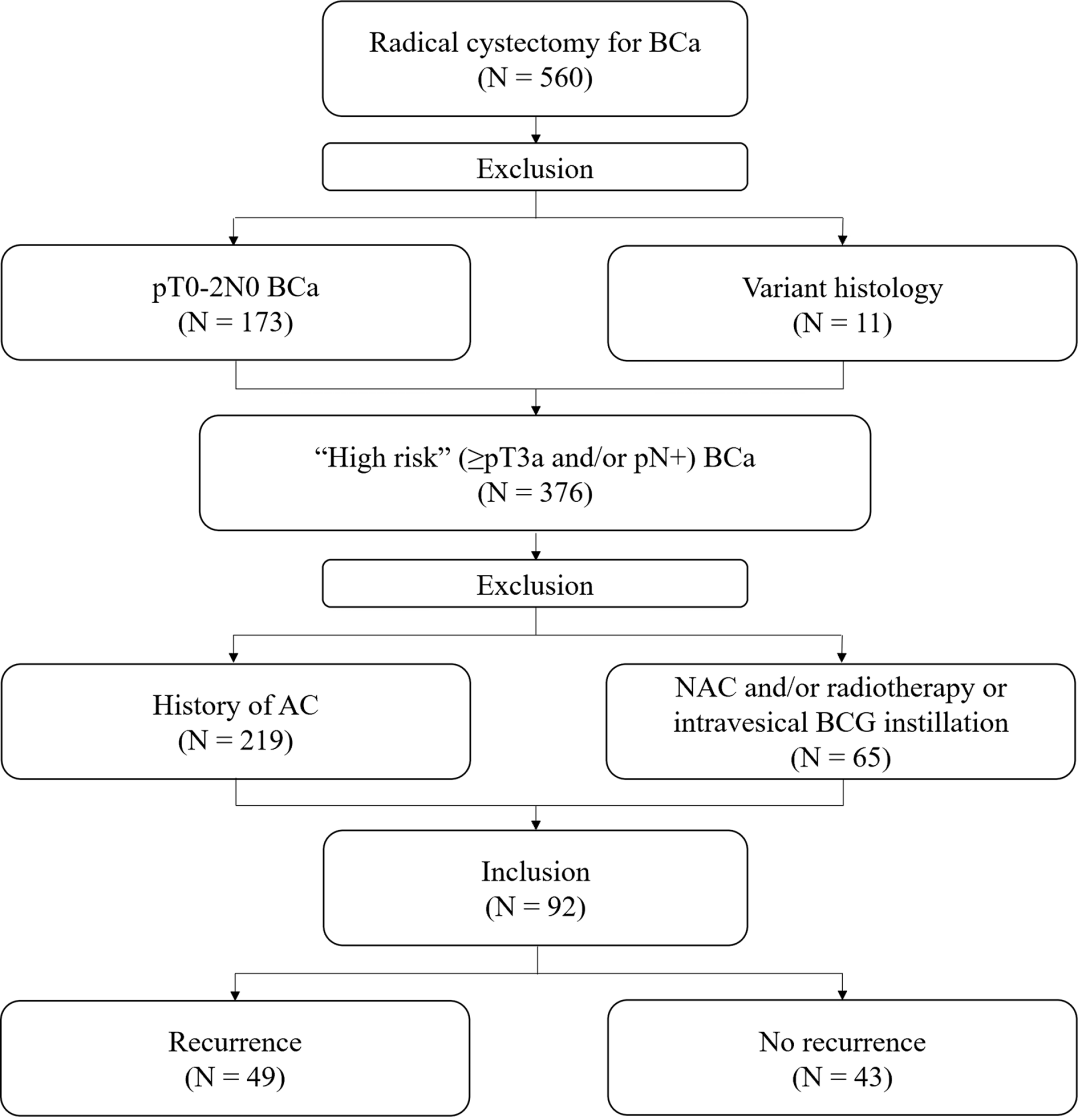
Flow chart of patient selection. BCa, bladder cancer; BCG, Bacillus Calmette- Guérin; AC, adjuvant chemotherapy; NAC, neoadjuvant chemotherapy.

We retrospectively examined a prospectively collected database of 560 patients who underwent RC for BCa between April 2014 and December 2019 by a single urologic surgeon. From the cohort, we excluded patients who were diagnosed with pT0-2N0 BCa following RC (n = 173) and who received NAC and/or radiation therapy or intravesical Bacillus Calmette- Guérin (BCG) instillation (n = 65). In addition, patients who had variant histology (n = 11) and those who received AC after RC (n = 219) were also excluded. Finally, 92 “high-risk” (≥pT3a and/or pN+) patients after RC without AC were selected for this study (**Figure 1**). All patients were preoperatively staged as cM0.

### Data Collection

Clinical and pathological characteristics of patients, including age at surgery, sex, pathologic T and N category, presence of *carcinoma in situ* (CIS), lymphovascular invasion (LVI), number of resected lymph nodes (LNs), status of surgical margin, and type of urinary diversion were identified from medical records.

Tumor recurrence was defined as local recurrence at the surgical bed or regional LNs, and/or distant metastasis. Recurrence-free survival (RFS) was calculated from the date of RC to the date of the first stated recurrence or the last follow-up date on which the patient was without tumor recurrence.

### Histologic Assessment

All RCs were conducted as open techniques and, in general, included removal of the prostate and seminal vesicle in men and removal of the ovaries and uterus in women. All patients received standard bilateral pelvic lymphadenectomy (15).

RC specimens were processed in formalin-fixed, paraffin-embedded sections. All specimens were reviewed by a pathologist specialized in genitourinary cancer. Pathologic staging and tumor grading were categorized according to the 2010 TNM classification of the American Joint Committee on Cancer (AJCC) and the 2004 World Health Organization (WHO)/International Society of Urologic Pathology consensus classification.

### Immunohistochemistry (IHC) Assay

PD-L1 on TIICs was measured using the VENTANA PD-L1 (SP142) rabbit monoclonal primary antibody (Ventana Medical Systems, Tucson, USA) with a fully automated IHC assay on the BenchMark ULTRA (Ventana Medical Systems, Tucson, USA) staining platform according to manufacturer protocols. The assay was optimized for the detection of PD-L1 in urothelial carcinoma, for which TIICs are predictive. The VENTANA PD-L1 (SP142) stain highlights a heterogeneous population of immune cells including lymphocytes, macrophages, dendritic cells, and granulocytes. In our study, most immune cells are blood-origin lymphocytes, and some granulocytes have been identified.

Briefly, formalin-fixed, paraffin-embedded tissue sections were cut in widths of 1.5 μm. After deparaffinization, antigen retrieval was performed using cell conditioning reagent 1 (Ventana Medical Systems, Tucson, USA). After primary antibody incubation at 37°C for 32 minutes, the Ultra View DAB Detection Kit (Ventana Medical Systems, Tucson, USA) was used for visualization. The slides were washed in distilled water, counterstained with hematoxylin (12 minutes) and bluing reagent (4 minutes), dehydrated in a descending order of alcohols, cleared in xylene, and coverslipped with Tissue-Tek mounting medium (Sakura Finetek Japan, Tokyo, Japan).

### Quantification of PD-L1 Expression in TIIC

**Figure 2:**
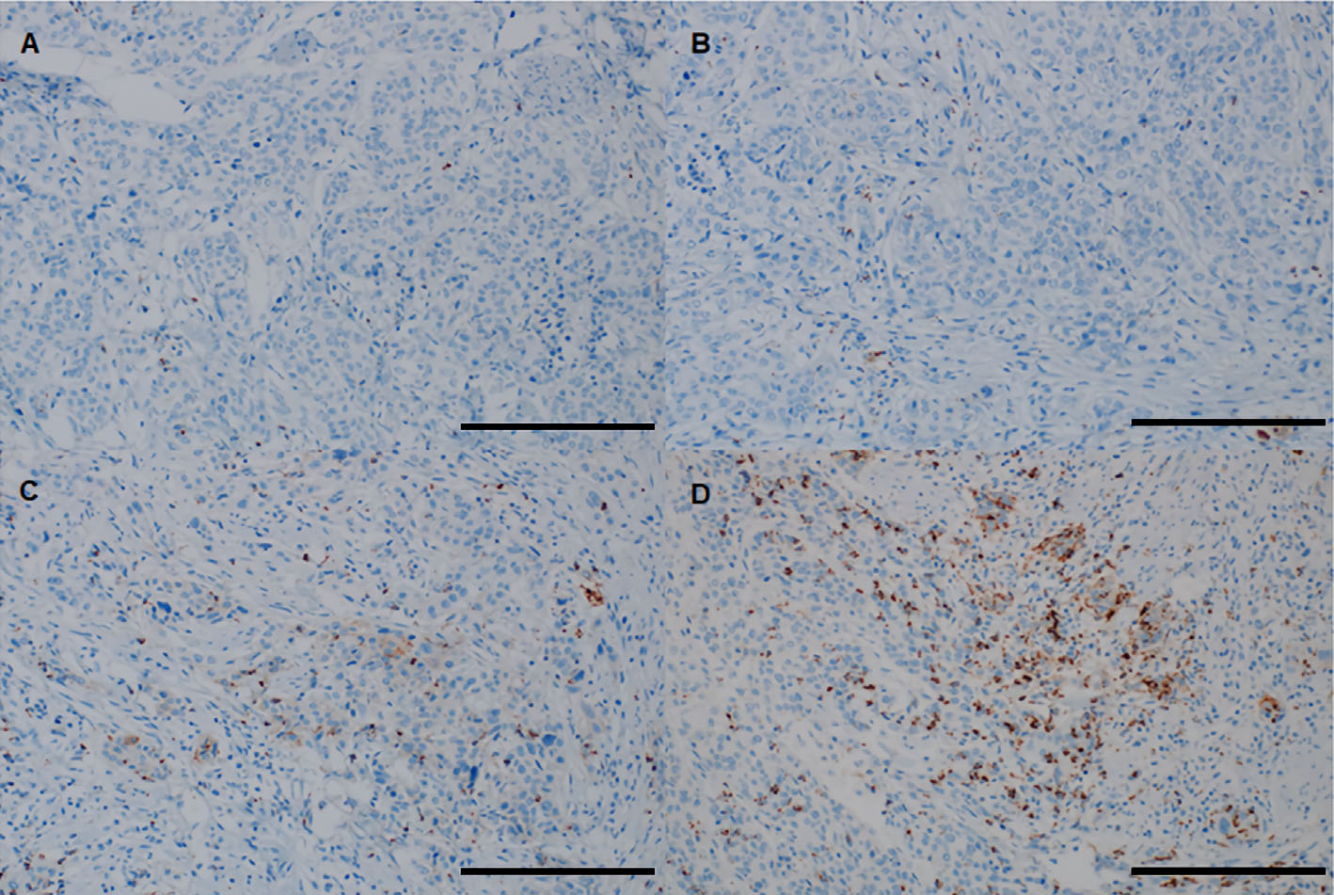
Representative images of PD-L1 on TIICs using the VENTANA (SP-142) immunohistochemistry assay in “high-risk” patients with bladder cancer. **(A)** IC0 (< 1% of tumor area covered by PD-L1 on TIICs), **(B)** IC1 (≥ 1% and <5% of tumor area covered by PD-L1 on TIICs), **(C, D)** IC2/3 (≥ 5% of tumor area covered by PD-L1 on TIICs). All images are ×200 magnification (scale bar 100 μm). PD-L1, programmed death-ligand 1; TIIC, tumor-infiltrating immune cell.

The evaluating pathologist was blinded to the clinicopathological and recurrence data of the patients. Patients were divided into three groups according to the percentage (%) of the tumor area covered by PD-L1 on TIICs: IC0 (<1%), IC1 (≥1% and <5%), and IC2/3 (≥5%). In addition, using a 5% cutoff value, PD-L1 was dichotomized as negative (<5%) or positive (≥5%) for statistical analysis. Representative images of PD-L1 on TIICs are depicted in **Figure 2**.

### Follow-Up Protocol

Each patient was followed up according to recommendations and institutional protocols. Following RC patients were generally scheduled at one month postoperatively, then every three months for the first two years, every six months for the next three years, and annually thereafter. During the follow-up period, a physical examination with laboratory tests, urine analysis with cytology, chest x-ray, and computed tomography (CT) or magnetic resonance imaging (MRI) of the chest, abdomen, and pelvis were performed at every visit to identify tumor recurrence. Bone scintigraphy was performed when clinically indicated.

### Statistical Analysis

Descriptive statistics were obtained for demographic variables. Continuous variables are presented as median (range) or mean (standard deviation, SD), and categorical variables are expressed as absolute values (percentages). An independent *t*-test was used to compare quantitative variables, and Pearson’s chi-square test, Fisher’s exact test, or linear-by-linear association were used to compare categorical clinicopathologic characteristics. Kaplan–Meier survival analysis was used to illustrate RFS, and differences were assessed using the log-rank test. Multivariable Cox proportional hazard models were used to identify predictive factors associated with tumor recurrence. All statistical analyses were performed using IBM SPSS Statistics for Windows, version 23.0 (IBM Corp. Armonk, NY, USA). Two-tailed *P*-values < 0.05 were considered statistically significant.

## Results

### Patient Characteristics

The baseline clinicopathological characteristics of the 92 “high-risk” patients who underwent RC for BCa without AC are outlined in **Table 1**. Within the cohort, the median (range) age at RC was 72.0 (61.0 - 82.0) years, and the male to female ratio was 4.4:1. Pathological tumor category T3 was identified in 72 patients (78.3%) at the time of RC. The median (range) resected number of LNs was 24.5 (8.0 - 42.0), and LN involvement was demonstrated in 44 patients (47.8%). The proportions of PD-L1 IC0, IC1, and IC2/3 on TIICs was 21.7%, 23.9%, and 54.4%, respectively. When patients were divided according to tumor recurrence (Yes *vs*. No), pathologic T and N categories and PD-L1 on TIICs were significantly different (all *P* < 0.05). However, there were no significant differences in age at surgery, sex, concomitant CIS, LVI, number of resected LNs, surgical margin status, and type of urinary diversion between the two groups.

**Table 1:** Clinicopathologic characteristics of 92 “high-risk” patients following radical cystectomy without adjuvant chemotherapy.

Parameters	Total	Recurrence		*P*
		Yes	No	
No. of patients	92 (100.0)	49 (53.3)	43 (46.7)	
Age at surgery, years				0.672
Median (range)	72.0 (61.0–82.0)	72.0 (61.0–82.0)	72.0 (63.0–81.0)	
Mean (SD)	71.6 (8.2)	71.2 (8.9)	71.9 (7.6)	
Gender, n (%)				0.611
Male	75 (81.5)	39 (79.6)	36 (83.7)	
Female	17 (18.5)	10 (20.4)	7 (16.3)	
Pathologic T stage at RC, n (%)				0.007
pT3	72 (78.3)	33 (67.3)	39 (90.7)	
pT4	20 (21.7)	16 (32.7)	4 (9.3)	
Concomitant CIS at RC, n (%)				0.856
Yes	48 (52.2)	26 (53.1)	22 (51.2)	
No	44 (47.8)	23 (46.9)	21 (48.8)	
LVI at RC, n (%)				0.504
Yes	61 (66.3)	34 (69.4)	27 (62.8)	
No	31 (33.7)	15 (30.6)	16 (37.2)	
No. of resected LNs at RC				0.807
Median (range)	24.5 (8.0–42.0)	25.0 (9.0–40.0)	23.0 (8.0–42.0)	
Mean (SD)	24.7 (8.1)	25.0 (7.6)	24.5 (8.6)	
Pathologic N status at RC, n (%)				0.001
Negative	48 (52.2)	15 (30.6)	33 (76.7)	
Positive	44 (47.8)	34 (69.4)	10 (23.3)	
Surgical margin status, n (%)				0.255
Negative	77 (83.7)	39 (79.6)	38 (88.4)	
Positive	15 (16.3)	10 (20.4)	5 (11.6)	
Type of urinary diversion, n (%)				0.139
Ileal conduit	14 (15.2)	10 (12.2)	4 (9.3)	
Orthotopic neobladder	78 (84.8)	39 (87.8)	39 (90.7)	
PD-L1 score on TIICs in RC specimens				0.001
IC0 (<1%)	20 (21.7)	8 (16.4)	12 (27.9)	
IC1 (≥1% and <5%)	22 (23.9)	6 (12.2)	16 (37.2)	
IC2/3 (≥5%)	50 (54.4)	35 (71.4)	15 (34.9)	
Follow-up, months				0.574
Median (range)	30.5 (16.1-66.5)	27.8 (16.1-64.9)	31.3 (16.6-66.5)	
Mean (SD)	31.3 (12.5)	30.5 (13.0)	32.1 (12.0)	

SD, standard deviation; RC, radical cystectomy; CIS, carcinoma in situ; LVI, lymphovascular invasion; LN, lymph node; PD-L1, programmed death-ligand 1; TIIC, tumor-infiltrating immune cell.

### Association of Clinic-Pathological Characteristics With PD-L1 Expression

The associations between clinicopathological characteristics and PD-L1 on TIICs are presented in **Table 2**. In patients with positive LNs the proportions of PD-L1 IC0, IC1, and IC2/3 on TIICs was 15.9%, 13.6%, and 70.5%, respectively, and the rates were significantly different from those in patients with negative LNs (*P* = 0.011). However, there was no association between PD-L1 on TIICs and any remaining clinicopathological characteristics, including age, sex, tumor stage, concomitant CIS, LVI, surgical margin status, and type of urinary diversion.

**Table 2:** Association of programmed death-ligand 1 expression and clinicopathologic characteristics.

Parameters	Total	PD-L1 expression on TIICs			*P*
		IC0	IC1	IC2/3	
Age					0.625
<72.0	45	8 (17.7)	12 (26.7)	25 (55.6)	
≥72.0	47	12 (25.5)	10 (21.3)	25 (53.2)	
Gender					0.474
Male	75	15 (20.0)	17 (22.7)	43 (57.3)	
Female	17	5 (29.4)	5 (29.4)	7 (41.2)	
Tumor stage					0.628
pT3	72	15 (20.8)	16 (22.2)	41 (57.0)	
pT4	20	5 (25.0)	6 (30.0)	9 (45.0)	
Concomitant CIS					0.929
Yes	48	10 (20.8)	11 (22.9)	27 (56.3)	
No	44	10 (22.7)	11 (25.0)	23 (52.3)	
LVI					0.151
Yes	61	10 (16.4)	14 (23.0)	37 (60.6)	
No	31	10 (32.3)	8 (25.8)	13 (41.9)	
Lymph node positivity					0.011
Negative	48	13 (27.1)	16 (33.3)	19 (39.6)	
Positive	44	7 (15.9)	6 (13.6)	31 (70.5)	
Surgical margin status					0.199
Negative	77	15 (19.5)	17 (22.1)	45 (58.4)	
Positive	15	5 (33.3)	5 (33.3)	5 (33.3)	
Type of urinary diversion					0.840
Ileal conduit	14	3 (21.4)	4 (28.6)	7 (50.0)	
Orthotopic neobladder	78	17 (21.8)	18 (23.1)	43 (55.1)	

CIS, carcinoma in situ; LVI, lymphovascular invasion; PD-L1, programmed death-ligand 1; TIIC, tumor-infiltrating immune cell.

### Association of PD-L1 Expression With RFS

**Figure 3:**
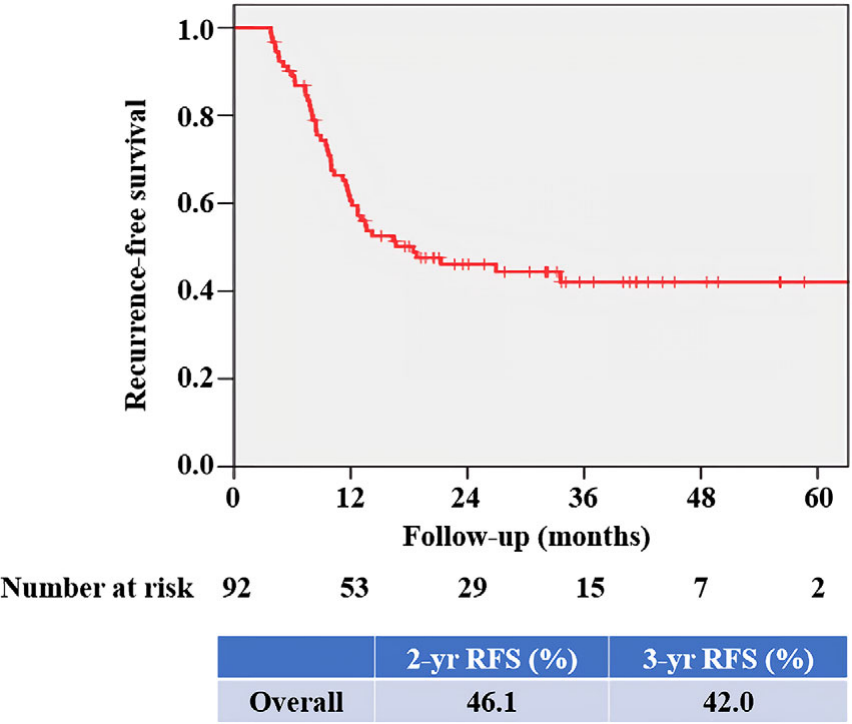
Kaplan–Meier survival curve for overall RFS. The 2- and 3-year overall RFS rates were 46.1% and 42.0%, respectively. RFS, recurrence-free survival.

**Figure 4:**
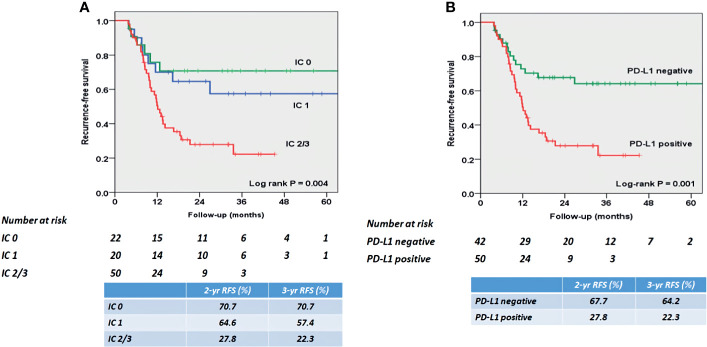
Kaplan–Meier survival curves for RFS according to **(A)** PD-L1 expression score, and **(B)** PD-L1 positivity on TIICs. **(A)** RFS was significantly shorter in patients with IC2/3 than in patients with IC0 (*P* = 0.005) and those with IC1 (*P* = 0.022). The 2- and 3-year RFS rates were 70.7% and 70.7% in patients with PD-L1 IC0, 64.6% and 57.4% in patients with PD-L1 IC1, and 27.8% and 22.3% in patients with IC2/3, respectively. **(B)** RFS was significantly shorter in patients with PD-L1 positive than in patients with PD-L1 negative (P = 0.001). The 2- and 3-year RFS rates were 67.7% and 64.2% in patients with PD-L1 negative. However, the 2- and 3-year RFS rates were 27.8% and 22.3% in patients with PD-L1 positive, respectively. PD-L1, programmed death-ligand 1; RFS, recurrence-free survival; TIIC, tumor-infiltrating immune cell.

At mean (SD) follow-up of 31.3 (12.5) months, tumor recurrence was identified in 49 patients (53.3%). The overall RFS rates estimated using the Kaplan–Meier method was presented in **Figure 3**. The 2- and 3-year overall RFS rates were 46.1% and 42.0%, respectively. However, when stratified according to PD-L1 on TIICs, RFS was significantly shorter in patients with IC2/3 than in patients with IC0 (*P* = 0.005) and those with IC1 (*P* = 0.022) (**Figure 4A**). Furthermore, when PD-L1 on TIICs was dichotomized using a 5% cutoff value, PD-L1 on TIICs significantly affected RFS (*P* = 0.001; **Figure 4B**) and using a 1% cutoff value, a significantly shorter RFS was also found in patients with positive PD-L1 on TIICs (*P* = 0.024). The 2- and 3-year RFS rates were 67.7% and 64.2% in negative PD-L1 on TIICs. However, the 2- and 3-year RFS rates were 27.8% and 22.3% in positive PD-L1 on TIICs, respectively.

The results of the Cox proportional hazard regression analysis of prognostic factors of tumor recurrence after RC without AC are summarized in **Table 3**. Multivariable Cox regression analyses showed that tumor category pT4 (hazard ratio [HR] = 2.23; 95% confidence interval [CI]: 1.278–2.3.896; *P* = 0.005), positive LNs (HR = 1.79; 95% CI: 1.083–3.232; *P* = 0.021), and positive PD-L1 on TIICs (HR = 2.03; 95% CI: 1.186–3.461; *P* = 0.010) were significantly associated with an increased risk of tumor recurrence.

**Table 3:** Cox proportional hazard regression analyses to predict tumor recurrence following radical cystectomy without adjuvant chemotherapy.

Variables	Univariable			Multivariable		
	HR	95% CI	*P*	HR	95% CI	*P*
Age						
<72.0	ref			ref		
≥72.0	1.01	0.989-1.031	0.305	1.02	0.993-1.042	0.215
Gender						
Male	ref			ref		
Female	1.21	0.617-2.381	0.577	1.15	0.553-2.402	0.704
Tumor stage						
pT3	ref			ref		
pT4	2.11	1.240-3.597	0.006	2.23	1.278-3.896	0.005
Concomitant CIS						
No	ref			ref		
Yes	1.34	0.866-2.212	0.201	1.38	0.842-2.243	0.253
LVI						
No	ref			ref		
Yes	1.08	0.512-2.234	0.839	1.10	0.586-2.055	0.773
Lymph node positivity						
Negative	ref			ref		
Positive	1.75	1.200-2.682	0.004	1.79	1.083-3.232	0.021
Surgical margin status						
Negative	ref			ref		
Positive	1.31	0.963-1.781	0.092	1.24	0.940-1.612	0.126
Type of urinary diversion						
Ileal conduit	ref			ref		
Orthotopic neobladder	0.95	0.550-1.628	0.843	0.85	0.385-1.885	0.691
PD-L1 on TIICs						
Negative	ref			ref		
Positive	1.71	1.028-2.855	0.039	2.03	1.186-3.461	0.010

CIS, carcinoma in situ; LVI, lymphovascular invasion; PD-L1, programmed death-ligand 1; TIIC, tumor-infiltrating immune cell.

## Discussion

In our study of 92 “high-risk” (≥pT3a and/or pN+) patients after RC without AC, tumor recurrence was confirmed in 49 patients (53.3%). When patients were stratified based on pathologic parameters, the RFS rate was significantly correlated with pathologic T and N categories and PD-L1 on TIICs. Positive PD-L1 on TIICs was closely associated with shorter RFS in patients with BCa after RC without AC. These results may help to establish treatment strategies for “high-risk” patients and advocate the use of adjuvant immunotherapy for “high-risk” patients with positive PD-L1 on TIICs. To the best of our knowledge, this is the largest study to evaluate the prognostic role of PD-L1 on TIICs in “high-risk” patients.

To date, the prognostic role of PD-L1 as a biomarker in BCa has been examined but showed inconsistent results owing to the use of different PD-L1 antibodies, location of PD-L1 measurement, and heterogeneous cutoff values to define positive PD-L1 expression (16–22). Therefore, to decrease the effect of heterogeneity between PD-L1 IHC assays, we only examined PD-L1 on TIICs that used the VENTANA assay with the SP 142 antibody. This assay was chosen because, in the past, atezolizumab was only reimbursed by the government for second-line treatment of metastatic BCa based on the result of VENTANA test. Furthermore, PD-L1 on TIICs was examined only in RC specimens derived from patients who had not received intravesical therapy and perioperative chemo-radiation therapy, which could have affected the outcomes (23, 24). In addition, we defined positive PD-L1 on TIICs based on a cutoff of 5%. As a result, positive PD-L1 on TIICs was detected in 54.4% (50/92) of patients.

In a study by Pichler et al. (16), 83 “high-risk” patients (≥pT3a and/or pN+ disease) who underwent RC without AC were included. In this study, positive PD-L1 on TIICs (defined as ≥1%) was identified in 61.4% (51/83) of patients, and the median RFS was significantly shorter in patients with positive PD-L1 on TIICs than in those with negative PD-L1 on TIICs (*P* = 0.015). They hypothesized that “high-risk” patients with positive PD-L1 on TIICs might be candidates for adjuvant immunotherapy following RC. In our study, 92 “high-risk” patients using the same inclusion criteria were analyzed and found that, using a 5% cutoff, PD-L1 on TIICs significantly affected RFS (*P* = 0.001). Furthermore, when a 1% PD-L1 cutoff was applied, a significantly shorter RFS was also found in patients with positive PD-L1 on TIICs (*P* = 0.024). Collectively, these results support the need for adjuvant immunotherapy following RC in “high-risk” patients with positive PD-L1 on TIICs.

In addition, we previously reported the prognostic role of PD-L1 on TIICs in patients treated with cisplatin-based AC following RC for patients with MIBC (25). In that study, 219 “high-risk” patients were included, and positive PD-L1 on TIICs (defined as ≥5%) was identified in 59.4% (130/219) of patients. We found that RFS was significantly poorer in patients with positive PD-L1 on TIICs than in those with negative PD-L1 on TIICs (*P* = 0.003). Taken together, these results indicate that positive PD-L1 on TIICs may be used as a prognostic biomarker in “high-risk” patients following RC irrespective of AC for selection of adjuvant immunotherapy.

Therefore, phase III trials to identify the efficacy of adjuvant immunotherapy following RC are ongoing. In a phase III IMvigor010 trial, there was no significant difference in disease-free survival (DFS) between atezolizumab and observation in high-risk patients following RC (19.4 months *vs*. 16.6 months, HR = 0.89; *P* = 0.2446) (12). However, in a phase III CheckMate-274 trial, nivolumab was identified as the first immune therapy to be used in the adjuvant setting that provided a clinically meaningful improvement in DFS in high-risk patients following RC both in the entire cohort (HR = 0.70; *P <*0.001) and in patients with PD-L1 ≥1% (HR = 0.53; *P <*0.001) (13). We are awaiting the results of a phase III AMBASSADOR trial (adjuvant pembrolizumab *vs*. observation) (14), and these trials will hopefully provide guidance for further treatment strategies.

For further application of the novel biomarker in future research, it is important to integrate with other available clinical predictors. The Vesical Imaging-Reporting and Data System (VI-RADS) using multiparametric MRI has been demonstrated to effectively differentiate among non-muscle invasive bladder cancer (NMIBC), MIBC and extravesical BCa (≥pT3) preoperatively (26–29). In addition, the VI-RADS scoring system might stratify patients according to earlier prediction of tumor response to treatment (30). Furthermore, biomarkers from blood samples such as circulating tumor cells (CTCs), surviving expressing CTCs and absolute basophil count are available for risk stratification in patients with NMIBC to predict recurrence and progression (31–34). Liquid biopsy biomarkers in urine and systemic combined inflammatory score also have the potential for diagnosis, prognosis and monitoring of oncologic outcome after treatment (35, 36). Collectively, integration with these potential biomarkers offers a more comprehensive decision-making tool for individualized treatment and an opportunity for future research.

Despite its potential clinical implications, this study is not devoid of several limitations that should be considered when interpreting the results. First, the retrospective study design performed at a single institution may have introduced inherent selection bias. Nonetheless, this study analyzed a prospectively accrued database and reflected real-world clinical experience. Second, to avoid the heterogeneity of IHC diagnostic assays, we only used the VENTANA assay with the SP 142 antibody to examine PD-L1 on TIICs. However, as PD-L1 staining has not been standardized, discrepancies due to different staining platforms, antibody clones, and scoring algorithms should be considered in the interpretation. Finally, due to a relatively short follow-up period, the correlation between PD-L1 on TIICs and cancer-specific survival or overall survival was not evaluated. Therefore, confirmation *via* a large, prospective validation study is required to corroborate the findings reported here.

In conclusion, in “high-risk” patients with BCa, PD-L1 is widely expressed on TIICs. Positive PD-L1 on TIICs was significantly associated with LN positivity and poorer RFS following RC without AC. Our results support the need for adjuvant immunotherapy in “high-risk” patients with positive PD-L1 on TIICs. Further prospective studies are needed to clarify the role of PD-L1 on TIICs as a biomarker in “high-risk” patients with BCa.

## Data Availability Statement

The raw data supporting the conclusions of this article will be made available by the authors, without undue reservation.

## Ethics Statement

The studies involving human participants were reviewed and approved by Institutional Review Board of Ewha Womans University Mokdong Hospital. Written informed consent for participation was not required for this study in accordance with the national legislation and the institutional requirements.

## Author Contributions

WS and DHL contributed to conception and design of the study. DHL organized the database. WS performed the statistical analysis. CUL wrote the first draft of the manuscript. All authors contributed to the article and approved the submitted version.

## Funding

This research was supported by the Basic Science Research Program through a National Research Foundation of Korea grant funded by the Ministry of Science, ICT & Future Planning (NRF-2018R1C1B6007678).

## Conflict of Interest

The authors declare that the research was conducted in the absence of any commercial or financial relationships that could be construed as a potential conflict of interest.

## Publisher’s Note

All claims expressed in this article are solely those of the authors and do not necessarily represent those of their affiliated organizations, or those of the publisher, the editors and the reviewers. Any product that may be evaluated in this article, or claim that may be made by its manufacturer, is not guaranteed or endorsed by the publisher.

## References

[1] Witjes JA Bruins HM Cathomas R Comperat EM Cowan NC Gakis G. European Association of Urology Guidelines on Muscle-Invasive and Metastatic Bladder Cancer: Summary of the 2020 Guidelines. Eur Urol (2021) 79(1):82–104. 10.1016/j.eururo.2020.03.055 32360052

[2] Apolo AB Grossman HB Bajorin D Steinberg G Kamat AM. Practical Use of Perioperative Chemotherapy for Muscle-Invasive Bladder Cancer: Summary of Session at the Society of Urologic Oncology Annual Meeting. Urol Oncol (2012) 30(6):772–80. 10.1016/j.urolonc.2012.01.012 PMC3524835 23218068

[3] Wosnitzer MS Hruby GW Murphy AM Barlow LJ Cordon-Cardo C Mansukhani M. A Comparison of the Outcomes of Neoadjuvant and Adjuvant Chemotherapy for Clinical T2-T4aN0-N2M0 Bladder Cancer. Cancer (2012) 118(2):358–64. 10.1002/cncr.26278 21717438

[4] Dash A Galsky MD Vickers AJ Serio AM Koppie TM Dalbagni G. Impact of Renal Impairment on Eligibility for Adjuvant Cisplatin-Based Chemotherapy in Patients With Urothelial Carcinoma of the Bladder. Cancer (2006) 107(3):506–13. 10.1002/cncr.22031 16773629

[5] Sternberg CN Bellmunt J Sonpavde G Siefker-Radtke AO Stadler WM Bajorin DF. ICUD-EAU International Consultation on Bladder Cancer 2012: Chemotherapy for Urothelial Carcinoma-Neoadjuvant and Adjuvant Settings. Eur Urol (2013) 63(1):58–66. 10.1016/j.eururo.2012.08.010 22917984

[6] Bellmunt J de Wit R Vaughn DJ Fradet Y Lee JL Fong L. Pembrolizumab as Second-Line Therapy for Advanced Urothelial Carcinoma. N Engl J Med (2017) 376(11):1015–26. 10.1056/NEJMoa1613683 PMC5635424 28212060

[7] Powles T Duran I van der Heijden MS Loriot Y Vogelzang NJ De Giorgi U. Atezolizumab Versus Chemotherapy in Patients With Platinum-Treated Locally Advanced or Metastatic Urothelial Carcinoma (IMvigor211): A Multicentre, Open-Label, Phase 3 Randomised Controlled Trial. Lancet (2018) 391(10122):748–57. 10.1016/S0140-6736(17)33297-X 29268948

[8] Gubin MM Zhang X Schuster H Caron E Ward JP Noguchi T. Checkpoint Blockade Cancer Immunotherapy Targets Tumour-Specific Mutant Antigens. Nature (2014) 515(7528):577–81. 10.1038/nature13988 PMC4279952 25428507

[9] van Wilpe S Gerretsen ECF van der Heijden AG de Vries IJM Gerritsen WR Mehra N. Prognostic and Predictive Value of Tumor-Infiltrating Immune Cells in Urothelial Cancer of the Bladder. Cancers (Basel) (2020) 12(9). 10.3390/cancers12092692 PMC7565173 32967190

[10] Horn T Grab J Schusdziarra J Schmid S Maurer T Nawroth R. Antitumor T Cell Responses in Bladder Cancer Are Directed Against a Limited Set of Antigens and Are Modulated by Regulatory T Cells and Routine Treatment Approaches. Int J Cancer (2013) 133(9):2145–56. 10.1002/ijc.28233 23625723

[11] Powles T Eder JP Fine GD Braiteh FS Loriot Y Cruz C. MPDL3280A (Anti-PD-L1) Treatment Leads to Clinical Activity in Metastatic Bladder Cancer. Nature (2014) 515(7528):558–62. 10.1038/nature13904 25428503

[12] Bellmunt J Hussain M Gschwend JE Albers P Oudard S Castellano D. Adjuvant Atezolizumab versus Observation in Muscle-Invasive Urothelial Carcinoma (IMvigor010): A Multicentre, Open-Label, Randomised, Phase 3 Trial. Lancet Oncol (2021) 22(4):525–37. 10.1016/S1470-2045(21)00004-8 PMC8495594 33721560

[13] UroToday. ASCO GU 2021: First Results From the Phase 3 CheckMate 274 Trial of Adjuvant Nivolumab vs Placebo in Patients Who Underwent Radical Surgery for High-Risk Muscle-Invasive Urothelial Carcinoma. Available at: https://https://www.urotoday.com/conference-highlights/asco-gu-2021/bladder-cancer/128091-asco-gu-2021-first-results-from-the-phase-3-checkmate-274-trial-of-adjuvant-nivolumab-vs-placebo-in-patients-who-underwent-radical-surgery-for-high-risk-muscle-invasive-urothelial-carcinoma-miuc.html (Accessed January 10, 2021).

[14] Clinical Trials. Testing MK-3475 (Pembrolizumab) After Surgery for Localized Muscle-Invasive Bladder Cancer and Locally Advanced Urothelial Cancer (AMBASSADOR). Available at: https://clinicaltrials.gov/ct2/show/NCT03244384 (Accessed January 10, 2021).

[15] Song W Yoon HS Kim KH Yoon H Chung WS Sim BS. Role of Bowel Suspension Technique to Prevent Early Intestinal Obstruction After Radical Cystectomy With Ileal Orthotopic Neobladder: A Retrospective Cohort Study. Int J Surg (2018) 55:9–14. 10.1016/j.ijsu.2018.04.044 29723678

[16] Pichler R Fritz J Lackner F Sprung S Brunner A Horninger W. Prognostic Value of Testing PD-L1 Expression After Radical Cystectomy in High-Risk Patients. Clin Genitourin Cancer (2018) 16(5):e1015–e24. 10.1016/j.clgc.2018.05.015 29960831

[17] Pichler R Heidegger I Fritz J Danzl M Sprung S Zelger B. PD-L1 Expression in Bladder Cancer and Metastasis and Its Influence on Oncologic Outcome After Cystectomy. Oncotarget (2017) 8(40):66849–64. 10.18632/oncotarget.19913 PMC5620140 28978000

[18] Bellmunt J Mullane SA Werner L Fay AP Callea M Leow JJ. Association of PD-L1 Expression on Tumor-Infiltrating Mononuclear Cells and Overall Survival in Patients With Urothelial Carcinoma. Ann Oncol (2015) 26(4):812–7. 10.1093/annonc/mdv009 25600565

[19] Stuhler V Maas JM Bochem J da Costa IA Todenhofer T Stenzl A. Molecular Predictors of Response to PD-1/PD-L1 Inhibition in Urothelial Cancer. World J Urol (2019) 37(9):1773–84. 10.1007/s00345-018-2538-6 30374610

[20] Wang B Pan W Yang M Yang W He W Chen X. Programmed Death Ligand-1 is Associated With Tumor Infiltrating Lymphocytes and Poorer Survival in Urothelial Cell Carcinoma of the Bladder. Cancer Sci (2019) 110(2):489–98. 10.1111/cas.13887 PMC6361576 30548363

[21] Powles T Walker J Andrew Williams J Bellmunt J. The Evolving Role of PD-L1 Testing in Patients With Metastatic Urothelial Carcinoma. Cancer Treat Rev (2020) 82:101925. 10.1016/j.ctrv.2019.101925 31785413

[22] Zhou TC Sankin AI Porcelli SA Perlin DS Schoenberg MP Zang X. A Review of the PD-1/PD-L1 Checkpoint in Bladder Cancer: From Mediator of Immune Escape to Target for Treatment. Urol Oncol (2017) 35(1):14–20. 10.1016/j.urolonc.2016.10.004 27816403

[23] McDaniel AS Alva A Zhan T Xiao H Cao X Gursky A. Expression of PDL1 (B7-H1) Before and After Neoadjuvant Chemotherapy in Urothelial Carcinoma. Eur Urol Focus (2016) 1(3):265–8. 10.1016/j.euf.2015.03.004 28723397

[24] Patel KR Taylor BL Khani F Guzzo TJ Scherr DS Ravishankar R. Impact of Neoadjuvant Chemotherapy on Concordance of PD-L1 Staining Fidelity Between the Primary Tumor and Lymph Node Metastases in Bladder Cancer. Urology (2019) 131:150–6. 10.1016/j.urology.2019.05.039 31201825

[25] Lee DH Jeong JY Song W. Prognostic Value of Programmed Death Ligand-1 Expression on Tumor-Infiltrating Immune Cells in Patients Treated With Cisplatin-Based Combination Adjuvant Chemotherapy Following Radical Cystectomy for Muscle-Invasive Bladder Cancer: A Retrospective Cohort Study. Onco Targets Ther (2021) 14:845–55. 10.2147/OTT.S291327 PMC7873022 33574678

[26] Del Giudice F Barchetti G De Berardinis E Pecoraro M Salvo V Simone G. Prospective Assessment of Vesical Imaging Reporting and Data System (VI-RADS) and Its Clinical Impact on the Management of High-Risk Non-Muscle-Invasive Bladder Cancer Patients Candidate for Repeated Transurethral Resection. Eur Urol (2020) 77(1):101–9. 10.1016/j.eururo.2019.09.029 31699526

[27] Woo S Panebianco V Narumi Y Del Giudice F Muglia VF Takeuchi M. Diagnostic Performance of Vesical Imaging Reporting and Data System for the Prediction of Muscle-Invasive Bladder Cancer: A Systematic Review and Meta-Analysis. Eur Urol Oncol (2020) 3(3):306–15. 10.1016/j.euo.2020.02.007 PMC7293940 32199915

[28] Del Giudice F Leonardo C Simone G Pecoraro M De Berardinis E Cipollari S. Preoperative Detection of Vesical Imaging-Reporting and Data System (VI-RADS) Score 5 Reliably Identifies Extravesical Extension of Urothelial Carcinoma of the Urinary Bladder and Predicts Significant Delayed Time to Cystectomy: Time to Reconsider the Need for Primary Deep Transurethral Resection of Bladder Tumour in Cases of Locally Advanced Disease? BJU Int (2020) 126(5):610–9. 10.1111/bju.15188 32783347

[29] Del Giudice F Pecoraro M Vargas HA Cipollari S De Berardinis E Bicchetti M. Systematic Review and Meta-Analysis of Vesical Imaging-Reporting and Data System (VI-RADS) Inter-Observer Reliability: An Added Value for Muscle Invasive Bladder Cancer Detection. Cancers (Basel) (2020) 12(10). 10.3390/cancers12102994 PMC7602537 33076505

[30] Panebianco V Pecoraro M Del Giudice F Takeuchi M Muglia VF Messina E. VI-RADS for Bladder Cancer: Current Applications and Future Developments. J Magn Reson Imaging (2020). 10.1002/jmri.27361 32939939

[31] Ferro M Di Lorenzo G Vartolomei MD Bruzzese D Cantiello F Lucarelli G. Absolute Basophil Count Is Associated With Time to Recurrence in Patients With High-Grade T1 Bladder Cancer Receiving Bacillus Calmette-Guerin After Transurethral Resection of the Bladder Tumor. World J Urol (2020) 38(1):143–50. 10.1007/s00345-019-02754-2 30993426

[32] Nicolazzo C Busetto GM Del Giudice F Sperduti I Giannarelli D Gradilone A. The Long-Term Prognostic Value of Survivin Expressing Circulating Tumor Cells in Patients With High-Risk Non-Muscle Invasive Bladder Cancer (NMIBC). J Cancer Res Clin Oncol (2017) 143(10):1971–6. 10.1007/s00432-017-2449-8 PMC11819121 28555356

[33] Nicolazzo C Busetto GM Gradilone A Sperduti I Del Giudice F Loreni F. Circulating Tumor Cells Identify Patients With Super-High-Risk Non-Muscle-Invasive Bladder Cancer: Updated Outcome Analysis of a Prospective Single-Center Trial. Oncologist (2019) 24(5):612–6. 10.1634/theoncologist.2018-0784 PMC6516110 30944184

[34] Busetto GM Ferro M Del Giudice F Antonini G Chung BI Sperduti I. The Prognostic Role of Circulating Tumor Cells (CTC) in High-Risk Non-Muscle-Invasive Bladder Cancer. Clin Genitourin Cancer (2017) 15(4):e661–e6. 10.1016/j.clgc.2017.01.011 28188046

[35] Ferro M La Civita E Liotti A Cennamo M Tortora F Buonerba C. Liquid Biopsy Biomarkers in Urine: A Route Towards Molecular Diagnosis and Personalized Medicine of Bladder Cancer. J Pers Med (2021) 11(3). 10.3390/jpm11030237 PMC8004687 33806972

[36] Ferro M Di Mauro M Cimino S Morgia G Lucarelli G Abu Farhan AR. Systemic Combining Inflammatory Score (SCIS): A New Score for Prediction of Oncologic Outcomes in Patients With High-Risk Non-Muscle-Invasive Urothelial Bladder Cancer. Transl Androl Urol (2021) 10(2):626–35. 10.21037/tau-20-1272 PMC7947442 33718065

